# Supplementary material for: Ultrafast neuromorphic photonic image processing with a VCSEL neuron
Source: Sci Rep. 2022 Mar 22;12:4874. doi: 10.1038/s41598-022-08703-1 (PMC8940934; doi:10.1038/s41598-022-08703-1)
Supplement: Supplementary file 1 — Supplementary Information. [file 41598_2022_8703_MOESM1_ESM.docx]

**Ultrafast Neuromorphic Photonic Image Processing with a VCSEL Neuron**

*Joshua Robertson*, Paul Kirkland, Juan Arturo Alanis, Matěj Hejda, Julián Bueno, Gaetano Di Caterina & Antonio Hurtado*

**Supplementary Information**

**Table of Contents**

1. **Vertical-Cavity Surface Emitting Lasers (VCSEL) Characterisation**
2. **Image Inputs and Optical Integration**
3. **Background Noise Variation**
4. **Spiking Neural Network (SNN) structure**
5. **Theoretical Analysis of Larger Kernel Operation**

**Vertical Cavity Surface Emitting Laser (VCSEL) Characterisation.** A single mode vertical-cavity surface-emitting laser (VCSEL) was used to create a photonic spiking processing system for image edge-feature detection. The VCSEL used in this work was a commercially available device operating at the telecom wavelength of 1550 nm. The device was tested and characterised prior to the operation demonstrated in the manuscript. Fig. S1 plots the VCSEL’s output power and lasing spectra at different bias currents when temperature stabilised at 293 K. Fig. S1a plots the L-I curve of the device measured at 293 K, showing a threshold current of I_th_ = 0.83 mA. When biased above threshold the device delivers continuous wave (CW) light emission. Fig. S1b shows the red shift of lasing spectra with increasing applied bias current. The VCSEL used in this work was a single-longitudinal and single-transverse mode laser source, which had two coexisting linear and orthogonally-polarised modes, referred to here as the orthogonally-polarised (λ*_x_*) and parallel-polarised (λ_y_) modes. The device operated with a dominant parallel λ_y_ mode and did not exhibit bias-induced polarisation switching across the measured operating parameters. At 4.0 mA the peak wavelength of the dominant λ_y_ mode was found at 1286.97 nm and the peak of the subsidiary λ*_x_* mode at 1287.11 nm. The corresponding wavelength separation between the two orthogonally-polarised modes for this device was measured as 0.136 nm (24.7 GHz). In this work injection of the image data input was made into the parallel λ_y_ mode of the device with injection polarisation matching the dominant mode of the VCSEL.


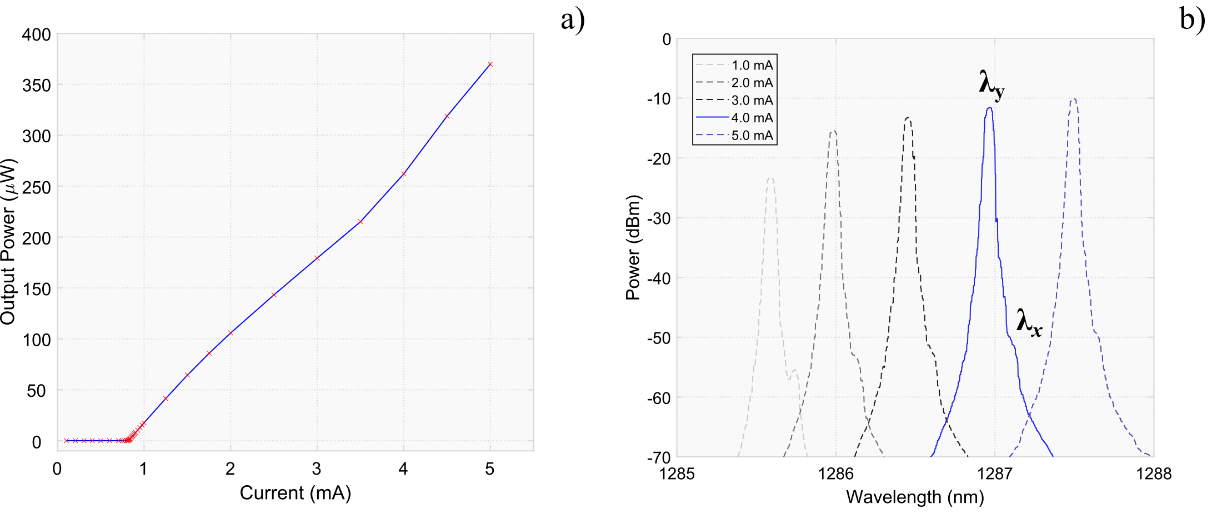


Fig. S1. Characterisation of the VCSEL. The output power was measured against bias current to determine the threshold of the device (a). The threshold current was equal to 0.83 mA. The lasing spectra of the device was recorded for increasing bias currents using an optical spectrum analyser (b). At the operating current of 4.0 mA the device demonstrated two orthogonal polarisation modes (λ_y_ and λ*_x_*) with peak wavelength of 1286.97 nm and 1287.11 nm respectively.

**Image Inputs and Optical Integration.** The photonic VCSEL neuron is responsible for the integration of optical inputs, the thresholding of their total contribution and the activation of fast spiking dynamics. Here, as described in Fig. 1 in the main article, image inputs are created by time-multiplexing the Hadamard product values of convolution operations, forming return-to-zero (RZ) encoded waveforms with bursts of positive and negative pulses. The RZ waveforms, or image inputs, are subsequently generated in an arbitrary waveform generator and encoded into the optical intensity of a tunable laser for injection into the VCSEL neuron. When the encoded injection enters the device, the inputs are integrated and the VCSEL neuron responds with fast spike events when its threshold for spike firing is exceeded. In this report the input pulses are generated at a rate of 12 GSa/s (1 sample per input), creating ~100 ps long pulses. The input pulses are grouped into fast ~700 ps bursts at the beginning of a configurable pixel window (equal to 3 ns in this work). Following the 3 ns window the pulse burst of the next pixel in the convolution process is generated. The generated image input is encoded into the optical injection entering the VCSEL neuron such that positive inputs produce negative intensity drops. Fig. S2a shows an example of encoded optical injection. The optical injection corresponds to the convolution of pixels 14-19 in row 13 of the printed “Digit 4” image with a 2x2 vertical kernel.


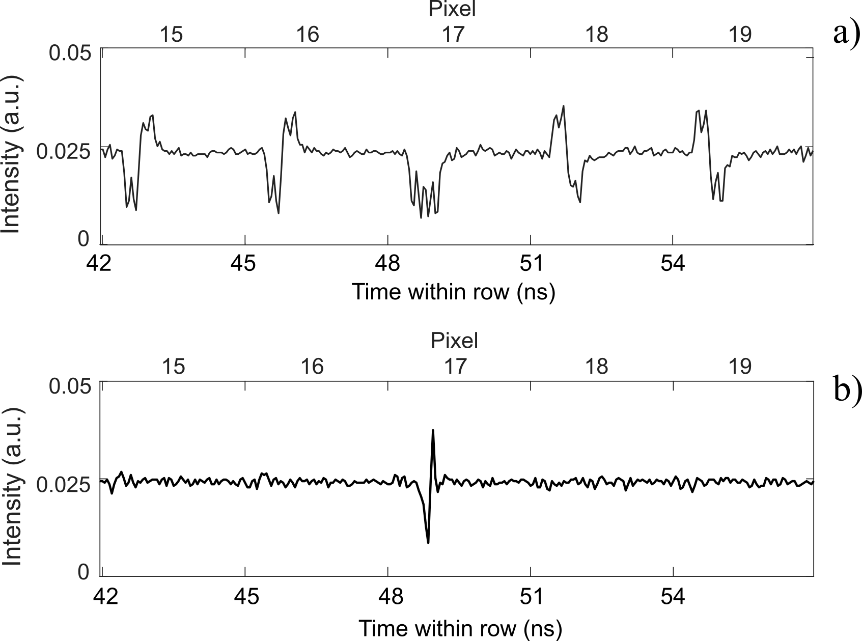


Fig. S2. Input-encoded injection and the subsequent VCSEL neuron response. The encoded optical injection, incident on the VCSEL neuron, is shown for the the convolution of pixels 14-19 in row 13 of the printed “Digit 4” with a 2x2 vertical kernel (a). The VCSEL neuron responds to the incoming signal by firing a spiking event for a target feature detection (pixel 17) (b).

As expected, the encoded optical injection takes the form of sequential bursts of positive and negative pulses. When a mismatch between kernel and local pattern descriptor (selected pixels) is present, combinations of positive and negative pulses occur within the same burst, as in the case of pixels 15,16,18 and 19 (see Fig. S2a). These inputs, when integrated by the VCSEL neuron, produce a low energy contribution to the activation threshold, subsequently failing to activate a spiking response in the recorded VCSEL neuron output shown in Fig. S2b. In contrast, when a kernel matches the local pattern descriptor, the injection is encoded with 4 negative inputs, as shown in pixel 17. Once injected into the neuron, the 4 negative inputs combine to produce a change in injection power that crosses the threshold for spike activation. This subsequently triggers an excitable spike at the output of the VCSEL neuron, as shown in Fig S2b. Following the successful or unsuccessful activation of spiking responses the system is reset during the remainder of the 3 ns window. This approach to input encoding and optical integration is used throughout this work including each demonstration of edge detection with the VCSEL neuron and the initial photonic hardware layer of the SNN used for the classification of MNIST hand-written digit (HWD) images.

**Background Noise Variation.**  As described in the main article, the edge detection performance of the system was tested on the printed “Digit 4” image for increasing source image noise. In addition to testing increasing global pixel noise, we have tested performance on source images with increasing background noise. To implement background noise in source images the intensity of each white pixel was varied randomly between its current value (-1) and a percentage (%) of the maximum intensity value (1). For a background noise of 100% white pixels could vary randomly between white (-1) and black (1). The source image was implemented with 0%, 20%, 40%, 60% and 80% background noise. Figs. S3(a) and 4(b) analyse the case of increasing background pixel noise, revealing good overall resilience to noise. Initially, when no noise is added, the system responded revealing all edge-features in the image. Increasing the background noise to 20% (Figs. 4(a)-(b)), did not dramatically affect performance, decreasing slightly (25.9% reduction) the number of successful detections (firing of sub-ns spikes). Increasing the background noise to 40% and 60% we observed the number of detections reduced overall (57.7% and 79.8% less successful activations). Despite this, the image became unrecognisable, and performance further decreased as background noise reached 80%. These results show that without altering the threshold of the system, target features can still be recognised up to 40% background noise.


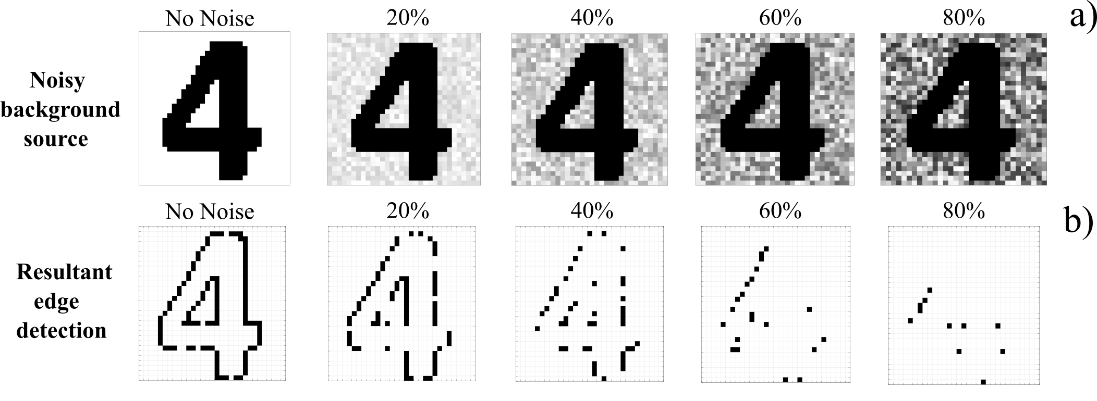


Fig. S3. Influence of source image noise on the edge-detection performance of the VCSEL-Neuron system. Noise was introduced to the printed “Digit 4” source image via the random variation of background-pixel intensity (a)-(b), and the random variation of global pixel intensity (c)-(d). Background pixels had their intensity values randomly varied by up to 80%, global pixel intensity was varied up to 20%.

**Spiking Neural Network (SNN) Structure.** A software implemented spiking neural network (SNN) is incorporated alongside a photonic VCSEL neuron spiking edge detection system to achieve the classification of MNIST handwritten digit images. As described in the main article, the optical spiking data generated by the experimental VCSEL neuron is fed to the software implemented SNN. The structure of the complete system is shown in Fig. S4.


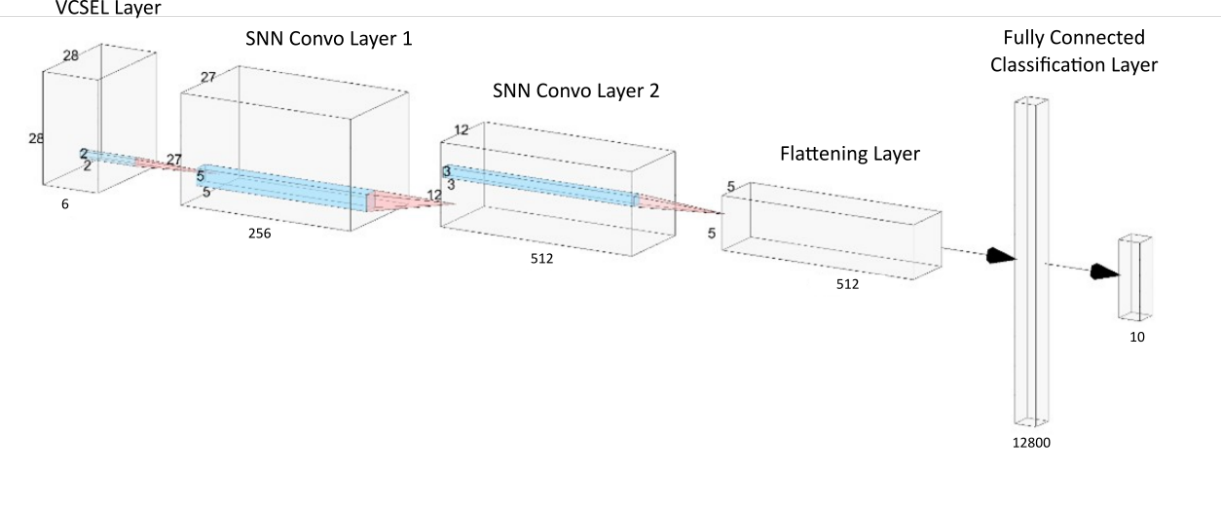


Fig. S4. The structure of Spiking Neural Network used in this work in combination with the VCSEL neuron photonic system for the classification of MNIST images. The first layer of the SNN is the experimental VCSEL neuron which performs spiking convolution with 2x2 kernel operators, creating a photonic spiking data stream. The software implemented SNN is fed the experimental data, subsequently performing convolution in 2 additional convolutional layers. The output of the second convolutional layer is flattened and passed to a 10 feature fully connected layer for classification.

The first layer of the SNN system is built experimentally with a single VCSEL neuron. This layer uses 6 2x2 kernel operators to yield edge-feature detection outputs in the form of fast optical spikes (as shown in Fig. 5 of the main article). The photonic spiking data is detected (with a >9.5 GHz bandwidth amplified photodetector) and recorded using a fast real-time oscilloscope. The recorded spiking time series are then fed into the software-implemented spiking neural network. The software implemented SNN made use hierarchical feature extraction and hence implemented a further two convolutional layers. The first SNN convolution layer created 256 feature maps using 5x5 kernel operators with a stride of 2x2, reducing the dimension of the feature maps to 12x12. The second convolution layer created 512 feature maps using 3x3 kernel operators again with a stride of 2x2, further reducing the dimension of the feature maps to 5x5. A flattening layer was used to prepare the network for the final 10 feature fully connected SoftMax layer which provided output probabilities for the final classification of the MNIST HWD image. The classification of 5000 MNIST HWD images were tested in this work and the recognition efficiencies are provided in Fig. 6 of the main article.

The network was constructed with the help of the Tensorflow and Keras libraries before being converted to a leaky integrate and fire system using the Nengo DL libraries. This method allowed backpropagation through gradient descent to be used to train the network weights prior to the conversion into a SNN. The completion of the training before SNN conversion also granted the use of well-established software training tools. In this work multiple training time steps were used before the final performance of the network was measured, as demonstrated in Fig. 6 of the main article. Overall, the method of converting a CNN to a SNN helped simplify implementation and training but did however influence the performance of the system. The average performance of the MNIST HWD classification reduced from 97.9% to 96.1% following the conversion to an SNN. This drop is performance is a trade off with the improvement to computational requirements, with the SNN using around 10% of that required by the CNN.

**Theoretical Analysis of Larger Kernel Operation.** To investigate further the potential of the neuromorphic image processing system of this work, convolution with larger (3x3) kernel operators was simulated using the Spin-Flip Model (SFM). The SFM equations (SI 1-5) were modified to include additional optical injection terms and were solved using the fourth order Runge-Kutta method:

 (SI 1)

$\frac{dN}{dt}=-\gamma_{N}\left[N\left(1+\left|E_{x}\right|^{2}+\left|E_{y}\right|^{2}\right)-\mu+in\left(E_{y}E_{x}^{*}-E_{x}E_{y}^{*}\right)\right]$ (SI 2)

$\frac{dn}{dt}=-\gamma_{s}n-\gamma_{N}\left[n\left(\left|E_{x}\right|^{2}-\left|E_{y}\right|^{2}\right)+iN\left(E_{y}E_{x}^{*}-E_{x}E_{y}^{*}\right)\right]$ (SI 3)

$F_{x}=-\sqrt{\frac{\beta_{sp}\gamma_{N}}{2}}\left(\sqrt{N+n}\xi_{1}+\sqrt{N-n}\xi_{2}\right)$ (SI 4)

$F_{y}=-i\sqrt{\frac{\beta_{sp}\gamma_{N}}{2}}\left(\sqrt{N+n}\xi_{1}+\sqrt{N-n}\xi_{2}\right)$ (SI 5)

In equations S1 1-5, subscripts *x* and *y* represent the subsidiary (orthogonally-polarised) and solitary (parallel-polarised) lasing modes of the VCSEL respectively. The field amplitudes of the subsidiary and solitary modes are represented by *E_x_* and *E_y_*. The total carrier inversion between conduction and valence bands is represented by *N,* with *n* representing the carrier inversion difference between spins of opposite polarity. *γ_a_* is the gain anisotropy (dichroism) rate, *γ_p_* is the linear birefringence rate, *γ_N_* is the decay rate of the carrier inversion and *γ_s_* is the spin-flip rate. *k* is the field decay rate, *α* is the linewidth enhancement rate and *μ* is the normalized pump current (where *μ*= 1 represents the VCSEL’s lasing threshold value). The injected image input, using here larger 3x3 kernel operators, is represented by *E_inj_* and the injection strength is controlled by *k_inj_*. The spontaneous emission noise *F_x_* and *F_y_* are calculated using the spontaneous emission strength *β_sp_* and two independent Gaussian white noise terms, *ξ_1,2_*, of zero mean and a unit variance. The angular frequency detuning is defined as Δ*ω_x_ = ω_inj_-ω_0_*, where the central frequency *ω_0_ =* (*ω_x_+ω_y_*)*/*2 lies between the frequencies of the subsidiary *ω_x_* = *ω_0_+αγ_a_-γ_p_* and the solitary mode *ω_y_* = *ω_0_+γ_p_-αγ_a_.* Δ*f = f_inj_-f_x_* is the frequency detuning between the injected field and the subsidiary mode, hence Δ*ω_x_ =* 2πΔ*f+αγ_a_-γ­_p_.* The following parameters were used to simulate the response of a VCSEL neuron: γ_p_ =128 ns^-1^, γ_a_ =2 ns^-1^, γ_N_ =0.5 ns^-1^, γ_s_ =110 ns^-1^, α =2, k =185 ns^-1^, k_inj_ =15 ns^-1^ and β_sp_ =10^-5^. *E_inj_* was introduced into the *x* polarisation mode with a frequency detuning of Δ*f =* -4 GHz.

Here, image inputs were generated using the same technique as that shown in Fig.1 of the main article. Each image input implements bursts of 9 input pulses (using a 3x3 kernel), one for each Hadamard product value. The input pulses were configured with pulse widths of 100 ps and with short pulse separations of 10 ps (to fit within the temporal integration window of the VCSEL neuron, equal to ~1 ns), within a configurable 3 ns pixel duration. As before, the simulated VCSEL is responsible for the integration of inputs and the activation of spiking responses for target features. The successful integration of a 9-burst input, and the activation of a fast spiking response, is shown in the numerically calculated result of Fig. S5. Here a burst of 9 inputs, is injected into the device producing the desired spiking response from the VCSEL, hence the integration of large input bursts can be theoretically performed.

Using an image from the MNIST handwritten digit database (Fig. S6 (a)), convolution with 8 3x3 kernel operators was numerically simulated (Fig. S6 (c)-(j)). The 8 3x3 kernel operators implemented integer weights, 1 for black and -1 for white, as shown at the bottom of Fig. S6. The results showed that each kernel operator successfully detected the target features within the MNIST image. The system performed the edge detection without the activation of false positives, indicating the effective integration of the larger bursts of 9 pulses (required for operation with 3x3 kernels) by the VCSEL neuron. This result reveals that the integrate-and-fire functionality of the VCSEL neuron can be utilized for larger bursts of input pulses (within the integration time window of the device) to detect specific features in complex source images. Overall, the 8 kernel reconstruction, shown in Fig. S6(b), reveals that complete edge detection can theoretically be performed with an integrate-and-fire VCSEL neuron.


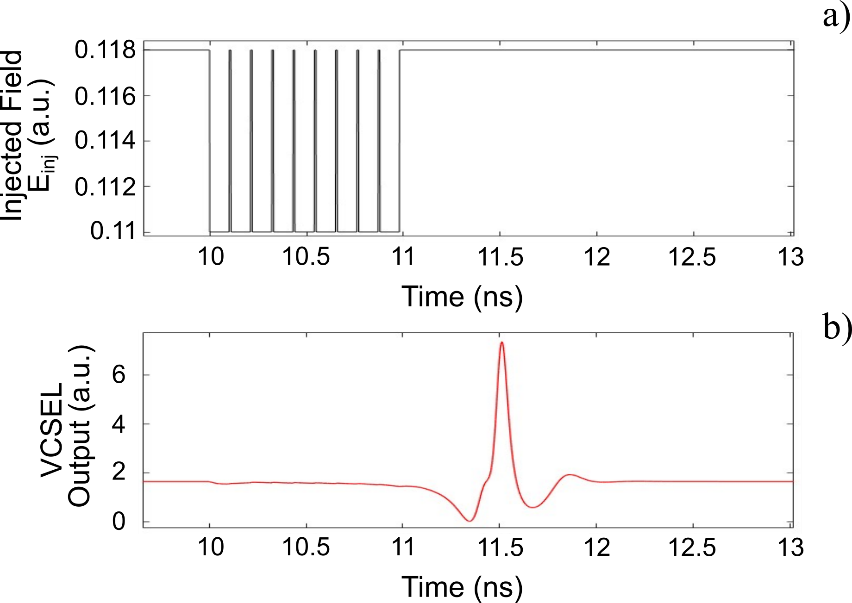


Fig. S5. Theoretical analysis of the operation of the VCSEL neuron under the injection of a large burst of 9-pulses (100 ps long and with 10 ps pulse separation). The injected field contains a burst of 9 input pulses corresponding to the detection of a target feature (a). Upon receiving this burst input, the VCSEL neuron fires a spike event (b), indicating the successful integration of the large burst and the detection of a target feature.


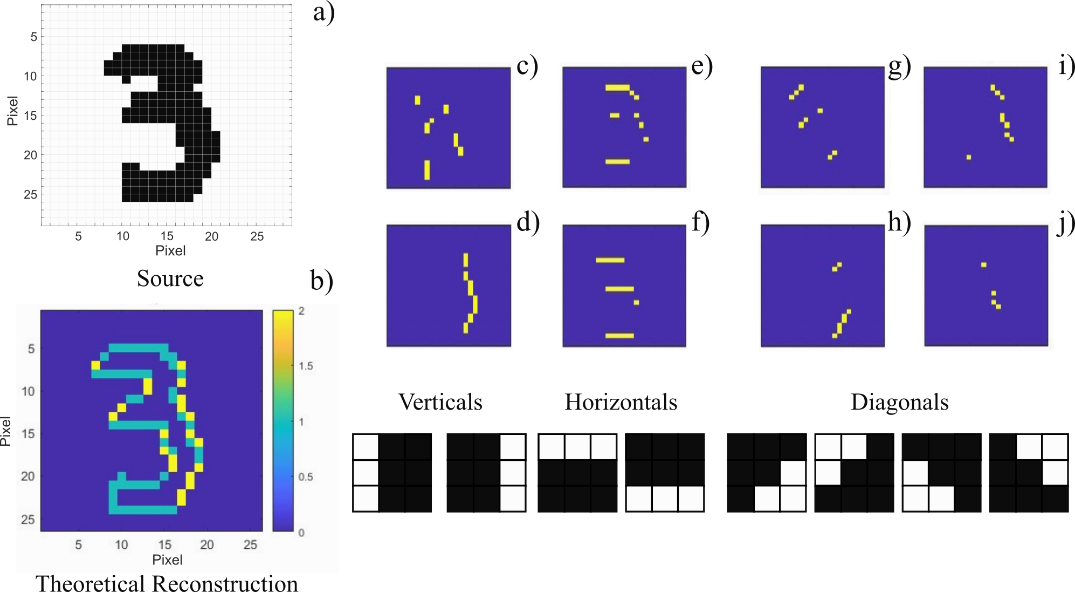


Fig. S6. Theoretical MNIST handwritten digit image edge-feature detection with a spiking VCSEL neuron using 3x3 kernel operators. The MNIST source image of a handwritten digit 3 (a) is operated on sequentially by 8 different 3x3 kernels (c)-(j). The two vertical (c)-(d), two horizontal (e)-(f), and four diagonal (g)-(j) kernel operators are shown in the insets at the bottom right side of the figure. (b) Reconstructed image combining the results obtained with the 8 kernel operators. This reveals all edges in the source image. Each plot in (b-j) is created by de-multiplexing the timeseries at the VCSEL neuron’s output, plotting pixels with a positive spike activation (recognition) in yellow/grey.
